# Supplementary material for: Strain-balanced type-II superlattices for efficient multi-junction solar cells
Source: Sci Rep. 2017 Jun 21;7:4012. doi: 10.1038/s41598-017-04321-4 (PMC5479808; doi:10.1038/s41598-017-04321-4)
Supplement: Supplementary file 1 — Supplementary Information. Strain-balanced type-II superlattices for efficient multi-junction solar cells [file 41598_2017_4321_MOESM1_ESM.pdf]

Supplementary Information

Strain-balanced type-II superlattices for efficient multi-junction solar cells

A. Gonzalo (1), A. D. Utrilla (1), D. F. Reyes (2), V. Braza (2), J. M. Llorens (3), D. Fuertes Marrón (4), B. Alén (3), T. Ben (2), D. González (2), A. Guzman (1), A. Hierro (1), J. M. Ulloa (1)*

(1) *Institute for Systems based on Optoelectronics and Microtechnology (ISOM), Universidad Politécnica de Madrid, Avda. Complutense 30, 28040 Madrid, Spain*

(2) *Departamento de Ciencia de los Materiales e IM y QI, Universidad de Cádiz, 11510 Puerto Real (Cádiz), Spain*

(3) *IMM-Instituto de Microelectrónica de Madrid (CNM-CSIC), Isaac Newton 8, PTM, E-28760 Tres Cantos (Madrid), Spain*

(4) *Instituto de Energía Solar (IES), Universidad Politécnica de Madrid, Avda. Complutense 30, 28040 Madrid, Spain*

* electronic mail: jmulloa@isom.upm.es

EQE normalization method

In order to compare the EQE of the different samples, the EQE of the bulk samples (samples bulk and GaAs) have been normalized to account for the fact that they have double amount of absorbing material than SL-samples.

As a first approximation, the internal quantum efficiency (IQE) is proportional to the absorptance

$$IQE(L) = 1 - e^{-\alpha L},$$

and at smaller thicknesses

$$IQE(L/N) = 1 - e^{-\frac{\alpha L}{N}} = 1 - \left(1 - IQE(L)\right)^{\frac{1}{N}},$$

The external quantum efficiency (EQE) is related to the IQE by means of the reflection coefficient

$$EQE(x) = (1 - R)IQE(x).$$

Combining these two expressions one can easily get a fair estimation of the EQE for a thinner thickness:

$$\boldsymbol{EQE\left(\frac{L}{N}\right) = (1 - R) \cdot \left(1 - \left[1 - \frac{EQE(L)}{(1 - R)}\right]^{1/N}\right),}$$

Here, we have assumed that there is no transmission through the sample and that the reflection is independent of the effective thickness. In our case, N=2 and R=0.33.

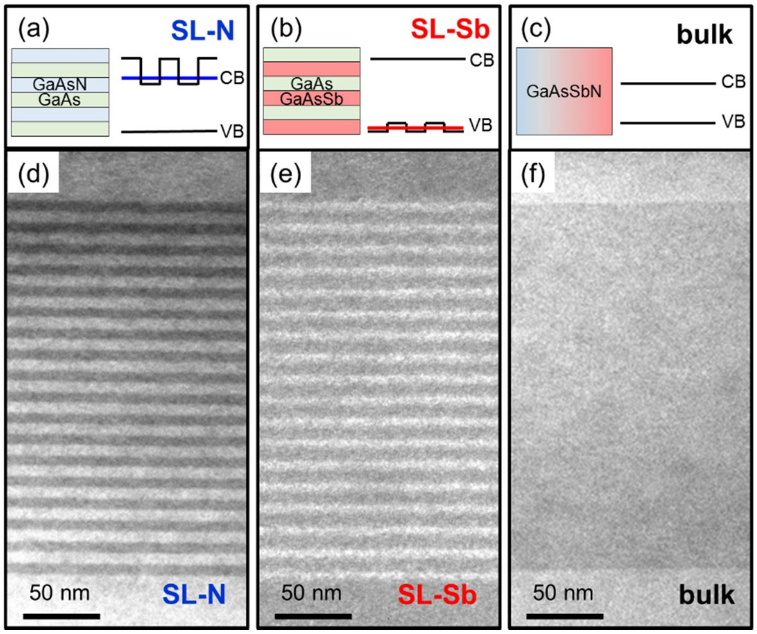

Figure S1. Epitaxial and band structure of 12 nm period ternary SL samples and bulk quaternary. (a,b,c) Sketch of the epitaxial layout and band alignment (not to scale) of the active region of the samples (a) *SL-N*, based on the stack of GaAsN and GaAs layers (b) *SL-Sb,* based on the stack of GaAsSb and GaAs layers (c) *bulk*, thick layer of the quaternary material. (d,e,f) Dark field 002 TEM images of samples (d) *SL-N* (e) *SL-Sb* and (f) *bulk.*

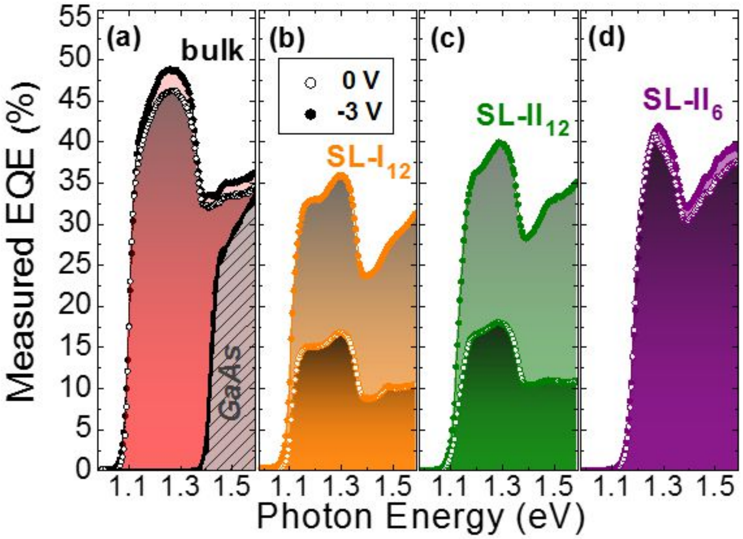

Figure S2. Measured external quantum efficiency of the solar cell devices. EQE spectra as measured at room temperature at 0 V (empty dots) and with a reverse bias voltage of -3 V (filled dots) from samples (a) *bulk* together with sample *GaAs* as a reference (b) *SL-I*$_{12}$ (c) *SL-II*$_{12}$ and (d) *SL-II*$_{6}$.

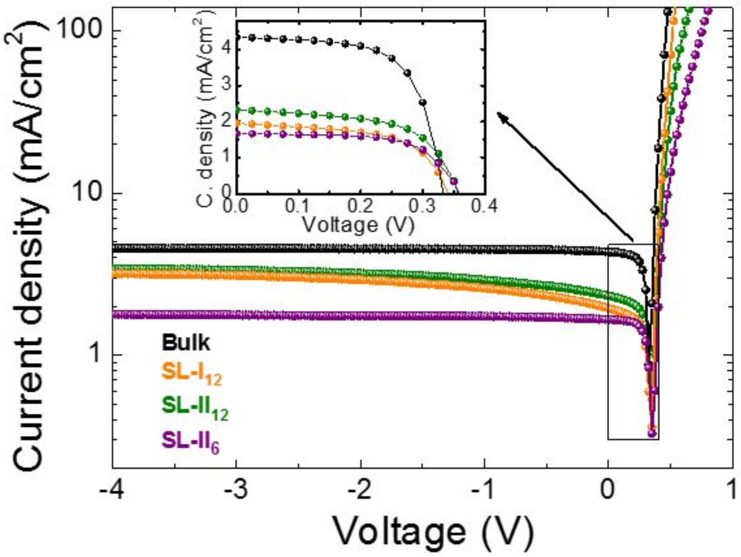

Figure S3. Current density-voltage (J-V) curves under monochromatic illumination of the solar cell devices. J-V curves of the samples *bulk*, *SL-I*$_{12}$, *SL-II*$_{12}$ and *SL-II*$_{6}$ under 1.2 eV (1033 nm) monochromatic illumination at room temperature. At -3 V current has already saturated in all cases, so this voltage can be considered to provide complete carrier collection conditions. The inset shows a magnification of the positive voltage part of the curves.

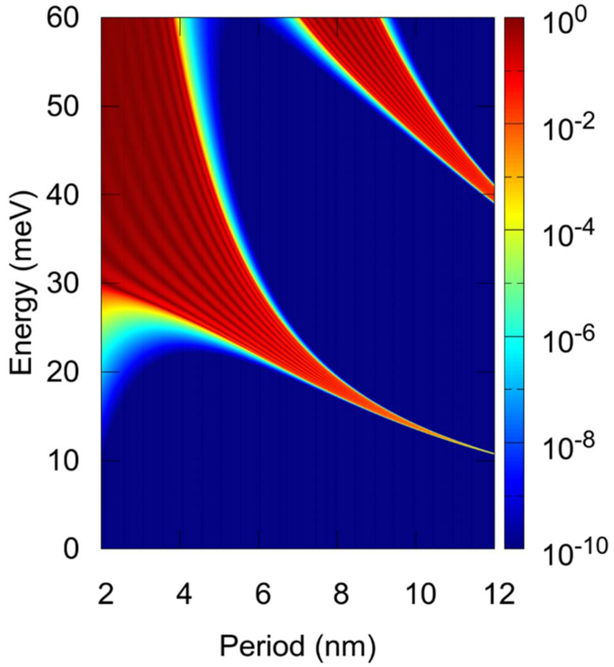

Figure S4. Transmission coefficient for holes below the GaAs bandgap energy as a function of the period thickness. Contour plot of the holes transmission coefficient for a 12 barrier/quantum well array as a function of period thickness. The height of the barrier is 70 meV and the effective mass 0.506 m_0, which are the parameters used to model the *SL-II* sample in Fig. 4b. The color bar is in log scale. The formation of electronically coupled Minibands for small periods becomes evident by the broadening of the high transmission coefficient region.

Table S1. Carrier lifetimes from time-resolved photoluminescence of samples *bulk*, *SL-I*, and *SL-II*. PL decay times in nanoseconds and their relative weights obtained from a multi-exponential fit to the experimental decay curves at the PL peak energy. The *bulk* and *SL-I* samples show a comparable behavior while in *SL-II* a much longer carrier lifetime appears.

	τ1 (ns) / w1 (%)	τ2 (ns) / w2 (%)	τ3 (ns) / w3 (%)
bulk	---	15.1 / 76.2	2.9 / 23.8
SL-I	---	15.5 / 78.0	3.3 / 22.0
SL-II	49.1 / 24.6	17.0 / 54.7	4.2 /20.7
